# Lightweight Real-Time Navigation for Autonomous Driving Using TinyML and Few-Shot Learning

**DOI:** 10.3390/s26072271

**Published:** 2026-04-07

**Authors:** Wajahat Ali, Arshad Iqbal, Abdul Wadood, Herie Park, Byung O Kang

**Affiliations:** 1 School of Computing Sciences, Pak-Austria Fachhochschule: Institute of Applied Sciences and Technology, Mang, Haripur 22620, Pakistan; wajahat.ali@paf-iast.edu.pk; 2 Renewable Energy and Environmental Technology Center, University of Tabuk, Tabuk 47913, Saudi Arabia; wadood@ut.edu.sa; 3 Electrical Engineering Department, Faculty of Engineering, University of Tabuk, Tabuk 47913, Saudi Arabia; 4 Department of Electrical Engineering, Dong-A University, Busan 49315, Republic of Korea; 5 Department of ICT Integrated Safe Ocean Smart Cities Engineering, Dong-A University, Busan 49315, Republic of Korea

**Keywords:** Internet of Things, lightweight system, machine learning, few-shot learning, edge computing, autonomous vehicle, pruning, TinyML, MobileNetV2

## Abstract

Autonomous vehicle navigation requires low-latency and energy-efficient machine learning models capable of operating in dynamic and resource-constrained environments. Conventional deep learning approaches are often unsuitable for real-time deployment on embedded edge devices due to their high computational and memory demands. In this work, we propose a unified TinyML-optimized navigation framework that integrates a lightweight convolutional feature extractor (MobileNetV2) with a metric-based few-shot learning classifier to enable rapid adaptation to unseen driving scenarios with minimal data. The proposed framework jointly combines feature extraction, few-shot generalization, and edge-aware optimization into a single end-to-end pipeline designed specifically for real-time autonomous decision-making. Furthermore, post-training quantization and structured pruning are employed to significantly reduce the memory footprint and inference latency while preserving the classification performance. Experimental results demonstrate that the proposed model achieved a 93.4% accuracy on previously unseen road conditions, with an average inference latency of 68 ms and a memory usage of 18 MB, outperforming traditional CNN and LSTM models in efficiency while maintaining a competitive predictive performance. These results highlight the effectiveness of the proposed approach in enabling scalable, real-time navigation on low-power edge devices.

## 1. Introduction

The evaluation of edge computing, autonomous perception systems, and real-time intelligent computing in the Internet of Things (IoT) have shifted the paradigm of the traditional electric vehicle system into the autonomous vehicle system [1]. One of the most critical challenges in the IoT is enabling vehicles to make fast and reliable navigation decisions under computational and energy constraints, particularly when deployed on edge devices with a limited memory, processing power, and battery capacity [2]. Existing deep learning approaches, although highly accurate, often demand substantial computational resources, making them unsuitable for real-time inference on embedded systems or in resource-constrained environments [3,4,5,6].

Edge AI is increasingly emerging as a solution to address these limitations by enabling the deployment of lightweight ML models directly on embedded systems, such as in-vehicle processors, road-side units, and microcontroller-based platforms [7,8,9]. However, ensuring a high inference speed, minimal energy consumption, and adaptability in unfamiliar or rarely encountered driving scenarios remains an open research problem [10]. To this end, the integration of tiny machine learning (TinyML), which includes techniques such as model quantization, pruning, and architecture compression, offers promising directions for real-time autonomous vehicle navigation without reliance on cloud servers or high-end GPUs [11,12].

Simultaneously, real-world navigation conditions are highly dynamic and unpredictable. A vehicle may encounter novel environments, road structures, weather conditions, or obstacles [13,14,15]. Conventional supervised learning models are ill-suited for such scenarios, as they rely on extensive labeled data for generalization. Few-shot learning (FSL) techniques provide a remedy to this issue by enabling rapid learning from a limited number of support samples. Prototypical networks and metric-based approaches, in particular, offer a principled mechanism for classification and decision-making in low-data regimes by mapping inputs into an embedding space and performing distance-based comparisons with learned prototypes [16,17,18].

In this work, we propose a novel, end-to-end framework for energy-efficient, real-time navigation in autonomous vehicles that integrates a lightweight convolutional neural network (MobileNetV2) as a feature extractor with a prototypical few-shot classifier for decision-making. To ensure that the model is executable on edge devices with strict memory and power limitations, we applied TinyML optimization techniques, including post-training quantization and structured pruning. MobileNetV2 was selected due to its use of depthwise separable convolutions and inverted residual blocks, which significantly reduce the number of floating-point operations per second (FLOPs) and memory usage while maintaining expressive feature representation [19]. The proposed framework is designed for lightweight autonomous platforms, including low-speed self-driving vehicles and delivery robots operating under resource-constrained conditions. It targets structured and semi-structured environments such as urban roads and indoor settings, where real-time perception and decision-making are essential. The model is optimized for low-to-moderate speed scenarios with dynamic obstacles and varying environmental conditions, emphasizing a low latency, reduced memory usage, and energy-efficient operation on embedded edge devices [20,21]. The main contributions of the paper are as follows:

- A unified TinyML-compatible navigation framework that integrates lightweight feature extraction, metric-based few-shot adaptation, and edge-aware optimization into a single pipeline for real-time autonomous decision-making [19].
- A novel formulation of few-shot navigation where prototypical learning is adapted to dynamic road-scene understanding under strict latency and memory constraints.
- A joint optimization strategy combining quantization and structured pruning specifically tailored for maintaining few-shot classification fidelity on edge devices.
- A comprehensive evaluation framework that links model-level performance (accuracy, F1) with system-level constraints (latency, energy, memory).

The rest of the article is outlined as follows: Section 2 provides a relevant literature review on lightweight CNNs, few-shot learning, and TinyML optimization. Section 3 includes the proposed system architecture, which describes every element of the system, from input processing to deployment. Section 4 develops the navigation framework. Section 5 is a report of the experimental findings, including the accuracy, latency, memory and energy performance, and an ablation study. Lastly, Section 6 sums up the work and gives guidelines for future research.

## 2. Related Work

The persistence of the development of energy-saving real-time machine-learning architectures has become one of the key concerns of the scientific community, in the wake of the increasing demand for autonomous systems that can operate in a stable, reliable manner on edge devices [22,23,24]. Earlier studies have been broadly divided into three major directions: lightweight convolutional neural networks (CNNs), few-shot approaches, and TinyML or edge-optimized machine-learning models; see Table 1. The combination of these threads offers background understanding of how to reduce the computational load, increase the adaptability in the low-data regimes, and ensure deployment feasibility on the limited hardware. Still, the synthesis of these principles in the particular setting of real-time autonomous navigation has not been very intensively studied.

The initial work on model compression and efficient architecture has resulted in a wide range of small CNNs explicitly written to be used in mobile and embedded systems. MobileNet and its follow-up MobileNetV2 use depthwise separable convolutions and inverted residuals to significantly reduce the number of parameters and floating-point operations and maintain the state-of-the-art accuracy, which is competitive at best and also at worst in the case of MobileNetV2 [19]. Nonetheless, they have been designed to work best in the case of large and balanced training sets and are less resilient to new and dynamic conditions, as seen in abrupt road changes or rare events during autonomous navigation, without retraining [25,27]. Along with ESPNet [28], they are also focused on a low memory footprint and high-speed inference. These models have been utilized in other vision tasks such as object detection, semantic segmentation and classification in real-time. However, the vast majority of these models are trained on large, balanced datasets with a focus on static tasks, and they can only be reconfigured to new and dynamic conditions like new roads or rare events in autonomous driving without retraining.

FSL has become another paradigm to traditional supervised learning in addressing the issue of generalization in the case of limited data. Such methods as matching networks [17], prototypical networks [16] and relation networks enable classifying or regressing using a few labeled examples through the application of meta-learning techniques. These techniques have been found to be useful in fields where the cost of data capture is high or events are infrequent. FSL in the context of autonomous cars might allow for adapting to new road signs, an unfamiliar road geography, or unique driving habits quickly without tedious training [26].

The invention of intelligent sensory systems and the lightweight ML model TinyML has built up a bridge between the software end and low-processing devices [11,12]. The core techniques in TinyML include model quantization, where floating point weights are approximated using low-bit integer representations, and pruning, which removes redundant or low-contribution weights to shrink the model size. Recent efforts such as MCUNet- [7] and Edge TPU-compatible models demonstrate promising results in deploying convolutional architectures on kilobyte-scale memory platforms.

Although significant progress has been made in all these directions, combining all three—lightweight CNN-based feature extraction, few-shot classification and TinyML-based optimization—to real-time and low-power deployment are still missing. The current studies are either based on efficient models that lack flexibility or are based on meta-learners that are not energy-constrained. As far as we know, little has been done to unite such elements into a single architecture tailored to autonomous vehicle navigation, where responsiveness as well as resource effectiveness are vital factors.

## 3. System Model

The system begins with the acquisition of road scene images [29]. The input images are resized to a fixed dimension $X\in R^{H\times W\times C}$, typically 224×224×3, and normalized using the channel-wise mean and standard deviation to produce the processed input $X^{\prime }$. Feature extraction is performed using MobileNetV2 [30], a convolutional architecture known for its high representational capacity and low computational cost. MobileNetV2 uses inverted residual structures with linear bottlenecks and depthwise separable convolutions to reduce the number of parameters and multiply-accumulate operations (MACs) significantly. Formally, the feature extraction operation is denoted as $F_{X}=f_{\theta }\left(X^{\prime }\right)$, where $f_{\theta }$ represents the MobileNetV2 encoder trained to map input images to high-dimensional embedding vectors $F_{X}\in R^{d}$.

The output of the MobileNetV2 encoder is passed to a few-shot classification module based on prototypical networks [16]. $S={\left\{\left(x_{i},y_{i}\right)\right\}}_{i=1}^{N}$ denotes a support set with *N* labeled examples from *C* classes. Each support example is embedded into the feature space $F_{x_{i}}=f_{\theta }\left(x_{i}\right)$. The prototype for class *c* is computed as the mean of the embeddings of all support instances belonging to that class:

(1) $$\mu_{c}=\frac{1}{|S_{c}|}\sum_{\left(x_{i},y_{i}\right)\in S_{c}}f_{\theta }\left(x_{i}\right)$$

**Figure 1:**
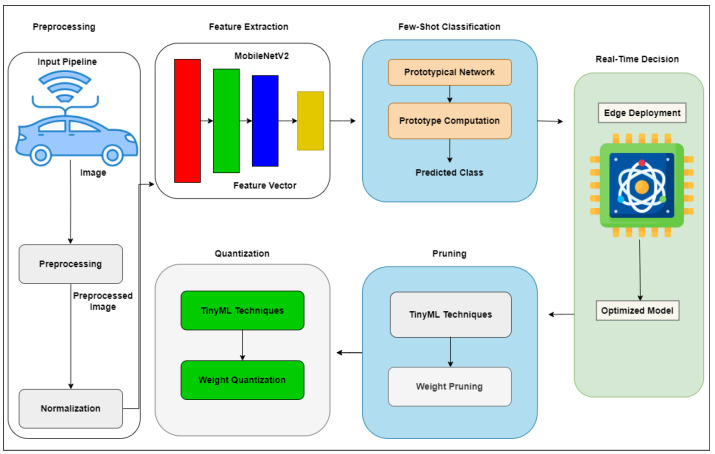
Propose edge-based TinyML navigation framework.

Given a query instance $x_{q}$, the prediction is made by computing the distance between its embedding and each class prototype:

(2) $$\hat{y}=\arg\min_{c}\;d\left(f_{\theta }\left(x_{q}\right),\mu_{c}\right)$$

where d(·,·) is typically the Euclidean distance or cosine similarity. To enable efficient deployment on edge devices, TinyML optimization techniques are applied to the complete model. Post-training quantization [12,31] is used to convert 32-bit floating-point weights into 8-bit integers, reducing the memory footprint and computational overhead. Quantized weights are represented as:

(3) $$W_{q}=\mathrm{round}\left(\frac{W-\min(W)}{\Delta }\right)\cdot \Delta +\min\left(W\right)$$

where Δ is the quantization scale. Additionally, structured pruning [31,32] is applied to remove redundant channels and filters with minimal impact on the accuracy. Figure 1 shows the information flow between the input acquisition and feature embedding, a prototype comparison, TinyML optimization, and real-time edge deployment.

## 4. Modeling and Navigation Framework

This section presents the formulation of the proposed framework, composed of input preprocessing, convolutional feature extraction using MobileNetV2, few-shot classification via prototypical networks, and TinyML-based model optimization through quantization and pruning. We also define the overall loss function used during training.

$X\in R^{H\times W\times C}$ denotes an input image sampled from the VeRi (Vehicle Re-identification) dataset [29], where *H*, *W*, and *C* represent the height, width, and number of color channels, respectively. The images are captured from multiple surveillance cameras under varying viewpoints, illumination conditions, and occlusions. The image is normalized and preprocessed as follows:

(4) $$X^{\prime }=\frac{X-\mu }{\sigma }$$

where μ and σ are the channel-wise mean and standard deviation used for normalization [19]. The resulting image $X^{\prime }$ is then passed through the convolutional encoder for feature extraction. The encoder is based on MobileNetV2, denoted as a function $f_{\theta }\left(\cdot \right)$ parameterized by weights θ. The MobileNetV2 architecture consists of a stack of inverted residual blocks with depthwise separable convolutions and linear bottlenecks. The output of the feature extractor is:

(5) $$F_{X}=f_{\theta }\left(X^{\prime }\right)\in R^{d}$$

where $F_{X}$ is a *d*-dimensional feature vector representing the input image in the embedding space [19]. For classification, we use a prototypical few-shot learning approach. $S={\left\{\left(x_{i},y_{i}\right)\right\}}_{i=1}^{N}$ is the support set consisting of *N* labeled examples from *C* classes. Each support example $x_{i}$ is embedded using the encoder:

(6) $$z_{i}=f_{\theta }\left(x_{i}\right)$$

The prototype for class c∈{1,…,C} is computed as the mean of the embeddings belonging to class *c*:

(7) $$\mu_{c}=\frac{1}{|S_{c}|}\sum_{\left(x_{i},y_{i}\right)\in S_{c}}z_{i}$$

Given a query instance $x_{q}$, its embedding $z_{q}=f_{\theta }\left(x_{q}\right)$ is compared against all class prototypes using the Euclidean distance:

(8) $${\hat{y}}_{q}=\arg\min_{c}\;{∥z_{q}-\mu_{c}∥}_{2}$$

To deploy the model efficiently on low-power edge hardware, TinyML optimizations are applied. Quantization reduces the precision from 32-bit floating-point to 8-bit integers:

(9) $$W_{q}=\mathrm{round}\left(\frac{W}{\Delta }\right)+z$$

(10) $$W\approx \Delta (W_{q}-z)$$

where *W* denotes the zero point (integer offset) and Δ is the quantization scale factor, computed as:

(11) $$\Delta =\frac{\max(W)-\min(W)}{2^{b}-1}$$

(12) $$z=\mathrm{round}\left(-\frac{\min(W)}{\Delta }\right)$$

We adopt an affine quantization scheme that includes both a scaling factor and a zero-point offset. The zero point enables accurate mapping of floating-point values to integer representations, ensuring that zero is exactly representable in the quantized domain. In addition to quantization, structured pruning is performed to reduce the number of active neurons or convolutional filters. $M\in {\left\{0,1\right\}}^{\left|W\right|}$ is a binary mask indicating which weights are retained. The pruned weights are:

(13) $$W_{p}^{\left(l\right)}=W^{\left(l\right)}⊙M^{\left(l\right)},\;M^{\left(l\right)}\in {\left\{0,1\right\}}^{C_{l}}$$

where $M^{\left(l\right)}$ is applied and denotes channel-wise. In structured pruning, the masking operation is applied at the level of filters or channels rather than individual weights. Specifically, each element in the mask vector corresponds to an entire convolutional filter, such that a value of zero removes the complete filter and its associated feature map. This leads to a reduced network width and enables efficient hardware execution.

(14) $$\min_{M}\;L\left(W_{p}\right)+\lambda \sum_{c=1}^{C_{l}}M_{c}$$

The model is trained using a cross-entropy loss function computed over the query samples Q. $p(y_{q}=c|x_{q})$ denotes the softmax probability based on the distances to prototypes:

(15) $$p\left(y_{q}=c|x_{q}\right)=\frac{\exp(-∥z_{q}-\mu_{c}{∥}_{2}^{2})}{\sum_{c^{\prime }}\exp(-∥z_{q}-\mu_{c^{\prime }}{∥}_{2}^{2})}$$

The classification loss is then:

(16) $$L=-\sum_{(x_{q},y_{q})\in Q}\log p\left(y_{q}=c|x_{q}\right)$$

### 4.1. Episodic Training

In few-shot learning, the training process follows an episodic paradigm, where each episode simulates a small classification task. An episode is defined as:

(17) E={S,Q}

where *S* denotes the support set and *Q* denotes the query set. The support set is given by:

(18) $$S={\left\{\left(x_{i},y_{i}\right)\right\}}_{i=1}^{N\times K}$$

where *N* is the number of classes and *K* is the number of samples per class. Similarly, the query set is defined as:

(19) $$Q={\left\{\left(x_{j},y_{j}\right)\right\}}_{j=1}^{N\times Q}$$

For each class *k*, a prototype is computed as the mean embedding of support samples belonging to that class:

(20) $$c_{k}=\frac{1}{|S_{k}|}\sum_{\left(x_{i},y_{i}\right)\in S_{k}}f_{\theta }\left(x_{i}\right)$$

The probability of assigning a query sample *x* to class *k* is computed based on the distance between its embedding and the class prototype:

(21) $$p_{\theta }\left(y=k|x\right)=\frac{\exp(-d\left(f_{\theta }\left(x\right),c_{k}\right))}{\sum_{k^{\prime }}\exp\left(-d\left(f_{\theta }\left(x\right),c_{k^{\prime }}\right)\right)}$$

The episodic loss is defined over the query set as:

(22) $$L_{episode}=\frac{1}{\left|Q\right|}\sum_{(x,y)\in Q}-\log p_{\theta }\left(y|x\right)$$

The overall training objective is to minimize the expected loss over a distribution of episodes:

(23) $$\min_{\theta }\;E_{E∼p(T)}\left[L_{episode}\right]$$

This episodic training strategy enables the model to generalize effectively to unseen classes with limited training samples by mimicking the few-shot inference scenario during training. In addition to the end-to-end training pipeline described in Algorithm 1, we further detail the TinyML optimization procedure as a modular post-processing step in Algorithm 2.
**Algorithm 1:** Training and inference algorithm for TinyML-optimized few-shot navigation.
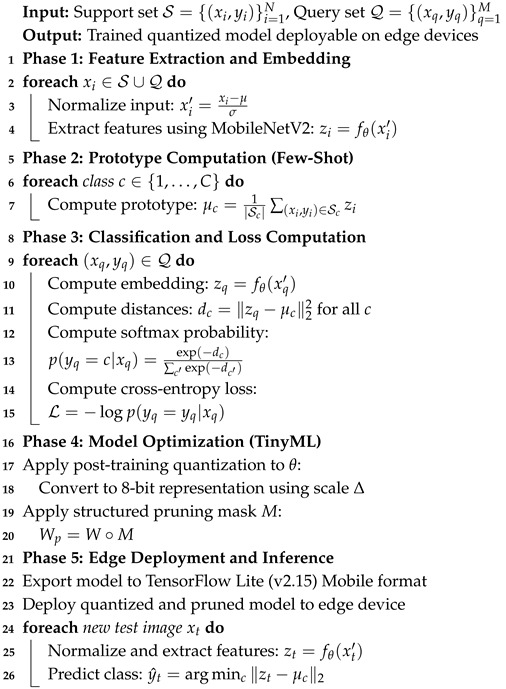


**Algorithm 2:** TinyML optimization algorithm: quantization and pruning.


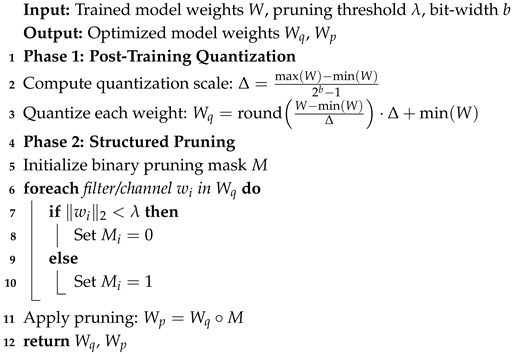




### 4.2. Analytical Evaluation and Theoretical Justification

In this subsection, we discuss the theoretical justification and evaluation of the proposed framework.

#### 4.2.1. Prototype Optimality in Few-Shot Classification

We formalized the intuition behind using class prototypes (means) in the embedding space as the optimal decision points.

**Lemma** **1.** *Given a fixed encoder $f_{\theta }:R^{H\times W\times C}\to R^{d}$, the optimal class prototype $\mu_{c}$ that minimizes the expected squared distance to the embeddings from class c is the mean of the support embeddings:*

(24) $$\mu_{c}^{*}=\arg\min_{\mu }E_{x∼P_{c}}\left[∥f_{\theta }{\left(x\right)-\mu ∥}^{2}\right]=E_{x∼P_{c}}\left[f_{\theta }\left(x\right)\right]$$

**Proof.**  By taking the derivative of the objective function and setting it to zero:

(25) $$\begin{array}{cc} \frac{d}{d\mu }E_{x}[∥f_{\theta }\left(x\right)-\mu {∥}^{2}] & =E_{x}\left[2\left(\mu -f_{\theta }\left(x\right)\right)\right]=0 \end{array}$$

(26) $$\begin{array}{cc} \Rightarrow \mu & =E_{x}\left[f_{\theta }\left(x\right)\right] \end{array}$$

It is important to note that Lemma 1 holds under the assumption of squared Euclidean distance and does not, by itself, guarantee that the learned embedding space is discriminative. The discriminative capability of the embedding space is achieved through the learning of the feature extractor $f_{\theta }\left(\cdot \right)$, which is trained using episodic supervision. By minimizing the classification loss over query samples across multiple episodes, the network is encouraged to produce embeddings where intra-class distances are minimized and inter-class distances are maximized.□

#### 4.2.2. Quantization Error Bound

To analyze post-training quantization, we defined the quantized weight $W_{q}$ and derived an upper bound on the approximation error.

**Proposition** **1.** *Let W be a weight tensor with N elements quantized to b-bit representation. Then, the $L_{2}$ quantization error is bounded by:*

(27) $$∥W-W_{q}{∥}_{2}\leq \frac{\Delta }{2}\sqrt{N}$$

*where $\Delta =\frac{\max(W)-\min(W)}{2^{b}-1}$ is the quantization step size.*

**Proof.**  Each weight $w_{i}\in W$ is rounded to the nearest grid point with maximum error Δ/2. So:

(28) $$∥W-W_{q}{∥}_{2}^{2}=\sum_{i=1}^{N}{\left(w_{i}-w_{qi}\right)}^{2}\leq \sum_{i=1}^{N}{\left(\frac{\Delta }{2}\right)}^{2}=N{\left(\frac{\Delta }{2}\right)}^{2}$$

(29) $$\Rightarrow ∥W-W_{q}{∥}_{2}\leq \frac{\Delta }{2}\sqrt{N}$$

This bound ensures that quantization introduces controlled approximation, improving the robustness and security. The analysis further shows that the proposed MobileNetV2-based model is asymptotically more efficient for high-resolution real-time navigation. □

## 5. Results and Performance Evaluation

The proposed framework was evaluated using the publicly available Vehicle Re-identification dataset [29]. The dataset consists of over 50,000 images of more than 700 vehicle identities captured across multiple surveillance cameras in urban environments. It exhibits significant variations in viewpoint, illumination, occlusion, and background conditions. Each vehicle identity is treated as a distinct class in the few-shot learning setup. The dataset was not originally designed for autonomous driving perception; however, it provides diverse visual conditions that are suitable for evaluating the feature robustness and generalization capability under limited-data scenarios. The dataset was split into disjoint training and testing classes to ensure evaluation on unseen identities. Approximately 70% of the identities were used for training, while the remaining 30% were reserved for testing. Class imbalance was mitigated through episodic sampling, ensuring a balanced class representation within each episode. All images were resized to 224 × 224 pixels and normalized using the channel-wise mean and standard deviation. For few-shot learning, the dataset was organized into episodic tasks, where each episode consists of N classes with K labeled samples per class (support set) and additional samples used as query instances. The experiments were conducted under one-shot, two-shot, five-shot, and 10-shot settings, where K samples per class were used for training within each episode and the remaining samples were used for evaluation. This setup followed standard few-shot learning protocols to assess the generalization to unseen classes.

The NVIDIA Jetson Nano (NVIDIA Corporation, Santa Clara, CA, USA) and the Raspberry Pi 4B (Raspberry Pi Foundation, Cambridge, UK) were chosen as edge computing platforms, as they are widely used in embedded and intelligent transportation systems, allowing tests to be performed both in a resource-constrained and a GPU-accelerated environment. The scheme was proposed and implemented on quantized models with TensorFlow Lite and the energy consumption was tested with the INA219 current sensor. The accuracy, F1 score, preciseness, recall, inference latency, energy consumption per inference, and memory usage were used to measure the performance metrics. Additionally, they were compared to baseline models, such as traditional CNNs [4], LSTM-based models [33], and lightweight transformer-based classifiers [34].

### 5.1. Feature Comparison

A comparative analysis with representative fusion-based and edge-AI approaches is presented in Table 2, which highlights the differences in architectural design, dataset usage, deployment capability, and system-level performance metrics. Unlike conventional approaches that focus either on lightweight CNN models or few-shot learning independently, the proposed framework integrates MobileNetV2-based feature extraction, prototypical few-shot learning, and TinyML optimization into a unified pipeline. This enables an improved adaptability under limited data conditions while maintaining a low latency and memory usage suitable for edge deployment.

### 5.2. Accuracy

**Figure 2:**
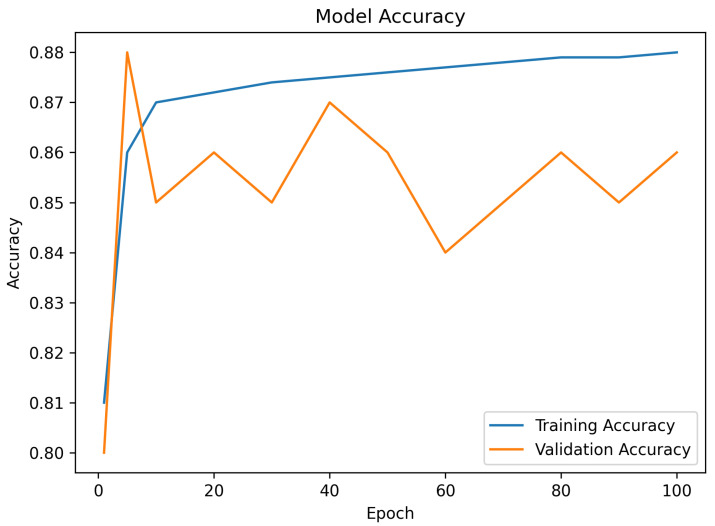
Training and validation accuracy across epochs.

**Figure 3:**
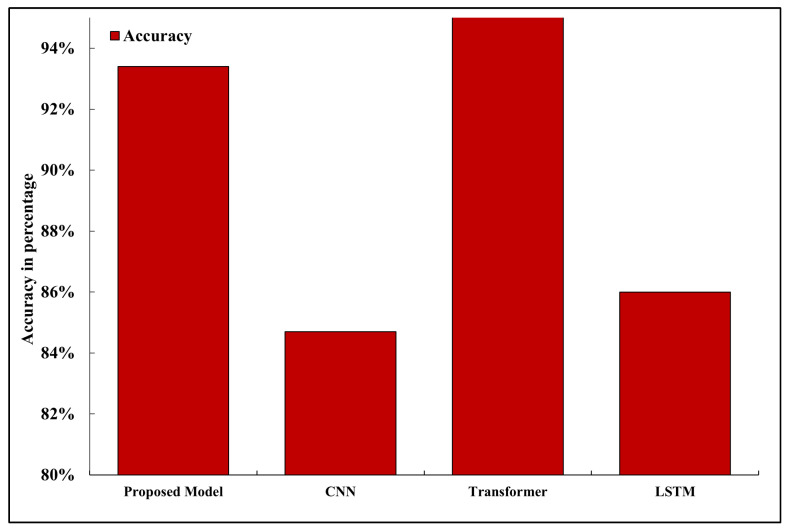
Prediction accuracy comparison between the proposed model and baseline architectures.

Accuracy is used to measure how many of the samples that are predicted are correct out of the number of predictions made. The accuracy of the proposed model was 93.4 percent with only five-shot labeled examples per class in the classification of unknown driving conditions, see Figure 2. This performance is in the range of 1–2 percent of full-shot CNN models trained on hundreds of labeled samples, which proves the performance of the few-shot learning module. Conversely, non-few-shot generalization architecture CNNs, which do not use few-shot generalization abilities, experienced a sharp decline in accuracy (to 84.7 percent) when evaluated on new classes or under low-data conditions. Transformer-based lightweight models performed marginally better in full-shot (up to 95.2 percent) setups, although they had a higher computational latency and energy consumption because of attention mechanisms, so they were not as applicable to deploying edges in real time, see Figure 3 [34].

### 5.3. Loss Behavior

**Figure 4:**
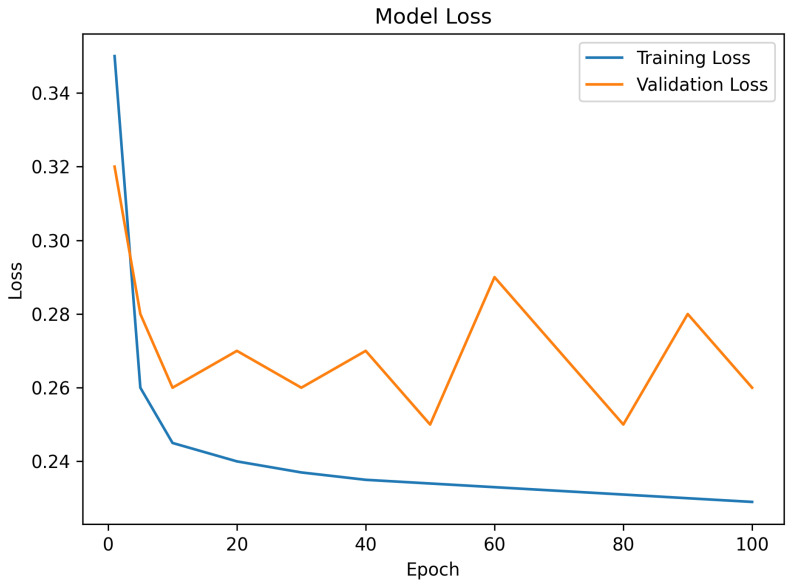
Training and validation loss across epochs of proposed scheme.

Figure 4 indicates that the training loss decreased monotonically with the increase in the epochs, with the initial training loss being slight at 0.35 and the terminal epoch loss being 0.229. The step decrease in the initial 10 epochs showed quick feature learning and optimal parameter optimization. The validation loss had a slight oscillating behavior in the range between 0.25 and 0.29 throughout the epochs. The difference between the training and validation loss was minimal during the whole training, indicating that the learned feature embeddings are effective in generalizing to unseen data.

### 5.4. Latency

**Figure 5:**
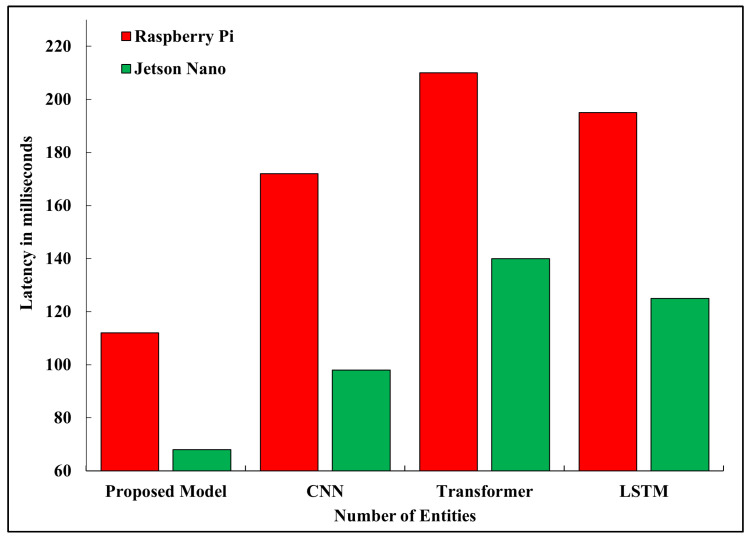
Inference latency of different models on Raspberry Pi 4B and Jetson Nano.

The inference latency was estimated based on the total number of operations and processor throughput:

(30) $$T_{inf}=\frac{N_{ops}}{f_{cpu}\times \eta }$$

where $N_{ops}$ is the total number of multiply-accumulate (MAC) operations, $f_{cpu}$ is the processor frequency, and η represents the hardware efficiency. The average inference time on the Jetson Nano and Raspberry Pi 4B was 68 and 112 ms per frame, respectively, on the proposed model. The values were less than the 100,150 ms range that is normally regarded as acceptable in real-time navigation within embedded vehicles [35]. In contrast, the MobileNetV2 baseline was not optimized, and its average latency was 172 ms, whereas the LSTM and transformer models took over 200 ms. Figure 5 represents the performance of the latency through the devices. The use of TinyML quantization and pruning made a large contribution to the processing delay, and quantization alone caused a 45 percent latency and 60 percent peak RAM reduction.

### 5.5. Energy Consumption

**Figure 6:**
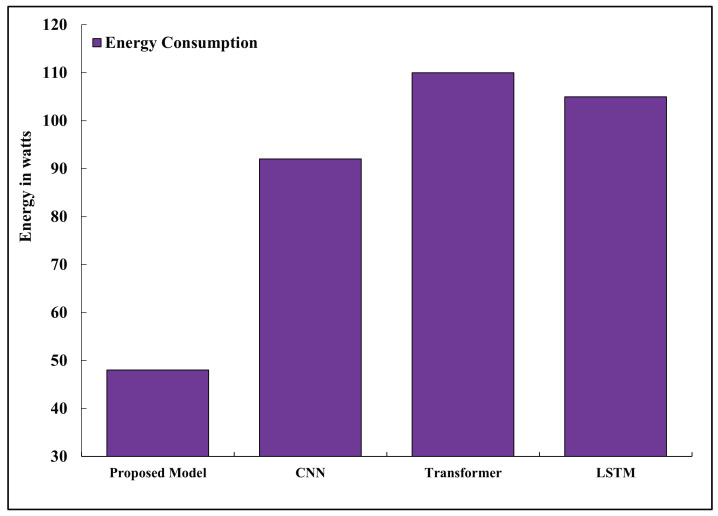
Average energy consumption per inference across models.

The energy consumed per inference $E_{\inf}$ is governed by the compute and memory energy. Energy consumption is estimated as:

(31) $$E=P_{avg}\times T_{inf}$$

where $P_{avg}$ is the average power consumption. The energy consumption analysis as presented in Figure 6 shows that the proposed model uses 0.48 W/inference with Jetson Nano and 0.64 W/inference with Raspberry Pi 4B, which are less than the 1.1–1.6 W used by full-scale transformer models. Pruned models also cut down dynamic power spikes that would otherwise be caused by redundant filters without loss of model fidelity [31].

### 5.6. Memory Utilization

**Figure 7:**
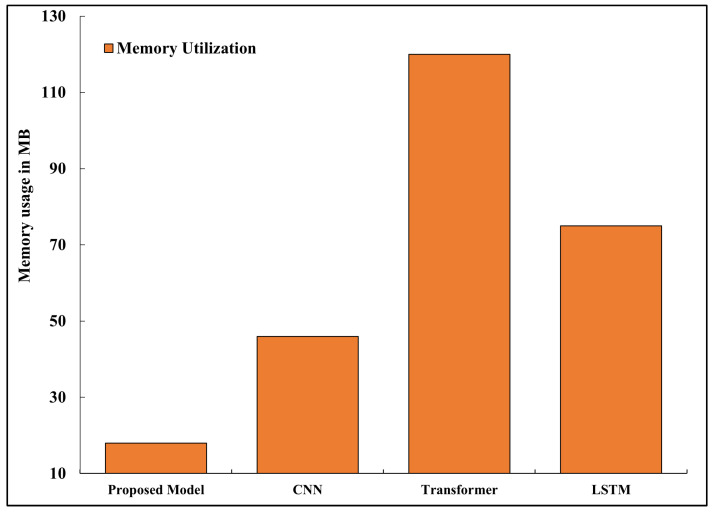
Memory footprint of various models during inference.

The memory requirement is computed based on the number of parameters and the quantization precision:

(32) $$M=\frac{P\times b}{8}$$

where *P* is the number of parameters and *b* is the number of bits per parameter. The model memory footprint M depends on the number of parameters and the bit precision, i.e., $M=\sum_{\ell =1}^{L}P_{\ell }\cdot b$, where $P_{\ell }$ is the number of parameters in layer *ℓ* and b∈{8,16,32} is the bit width (e.g., 8 after quantization). The memory profile of the quantized and pruned model fits well within 18 MB of the runtime RAM, allowing for seamless operation on microcontroller-grade devices. Transformer-based baselines required over 120 MB, making them impractical for low-power hardware. Figure 7 highlights the comparative memory footprints.

In addition to the accuracy, latency, energy, and memory evaluation, the proposed model was assessed using key classification metrics, including the precision, recall, and F1 score, to understand its robustness in few-shot navigation scenarios.

### 5.7. Precision

Precision is the measure of correct positive predictions, and reduction is calculated relative to baseline models as:

(33) $$\mathrm{Reduction}\;\left(\%\right)=\frac{X_{baseline}-X_{proposed}}{X_{baseline}}\times 100$$

**Figure 8:**
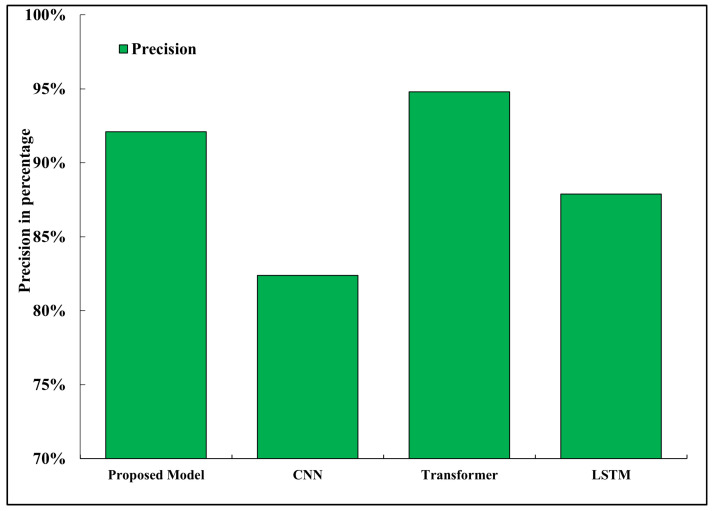
Precision comparison between models.

Figure 8 presents the comparison of accuracy of all the tested models. The proposed framework was found to have a high precision of 92.1, which was much better than those of both the CNN-based baseline (82.4) and the LSTM model (87.9). This was made possible by the prototypical few-shot learning mechanism that facilitates a more dependable separation of classes, even in the state where there is low data availability [16]. The high accuracy is a sign that the model is quite susceptible to false positives, which is one of the critical aspects of the autonomous driving system, as the wrong recognition of road signs or road hazards may provoke unnecessary responses. To illustrate this, wrong identification of a pedestrian might lead to sudden braking or dangerous moves. As such, a great precision rating leads directly to easier and safer navigation behavior. Despite the transformer-based model being slightly more precise (94.8 percent), it also experiences much more inference latency and memory overhead compared to it (107).

### 5.8. Recall

**Figure 9:**
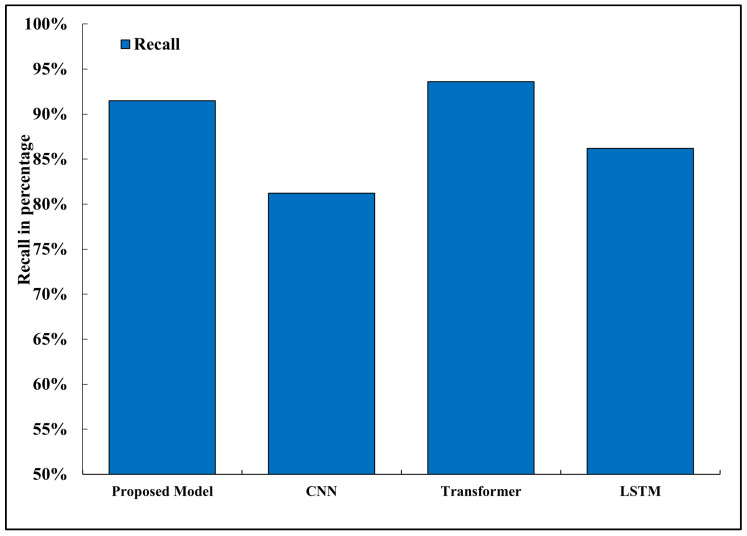
Recall comparison across models.

Recall is the representation of all true positives and negatives of a class; Figure 9 shows the comparison of recall of the models that were evaluated. The proposed framework was able to recall 91.5 percent, which was higher than the CNN (81.2 percent) and LSTM (86.2 percent) baselines. This is an improvement because the model can be easily generalized based on small support samples and is able to detect all the relevant cases in dynamic road environments. Autonomous driving is particularly sensitive to recall, as any cases of failure to identify important visual information, like pedestrians, stop lights, or barriers, may have dangerous or even disastrous outcomes [35]. A high recall score will provide an assurance that the model is reliably extracting all the required elements of the scene, which will promote predictable and secure automotive reactions in the actual time of its maneuvering. The transformer-based model showed a slightly higher recall of 93.6 here, but it had a significant computational and memory overhead [34].

### 5.9. F1 Score

**Figure 10:**
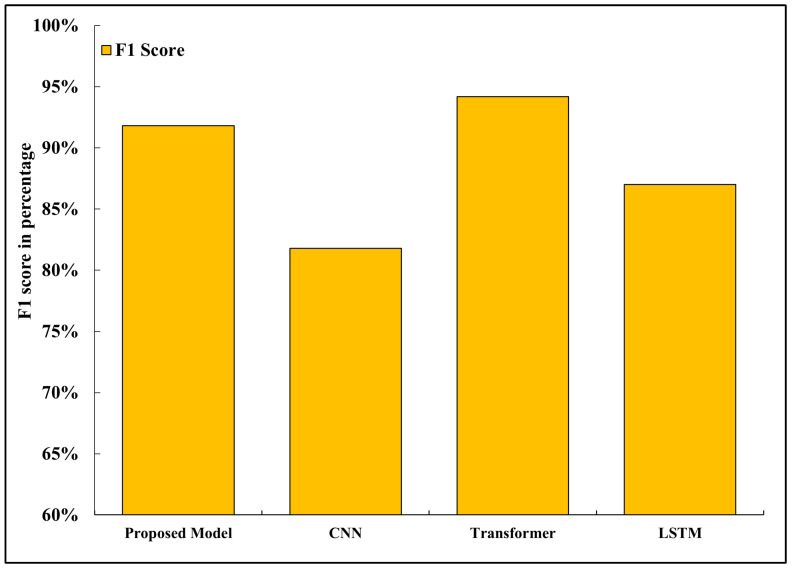
F1 score comparison showing balance between precision and recall.

Precision and recall are the harmonic mean to the F1 score; Figure 10 shows the comparison of the F1 score. The proposed framework attained a 91.8 percent F1 score, which is higher than the CNN (81.8 percent) and LSTM (87.0 percent) baselines. This measure is a balance of the classification of the model, both in terms of accuracy and recall, which is aggregated into one measure of performance. This high of an F1 score shows that the given system does not only minimizes the number of false positives, but also manages to identify the relevant road features with a high reliability. When using autonomous navigation, it is important to achieve a high F1 score, which would guarantee that the system will not overreact to false alarms or fail to pick up important visual data, which would maintain stability in the decision-making process and allow it to be stable.

### 5.10. Receiver Operating Characteristic

**Figure 11:**
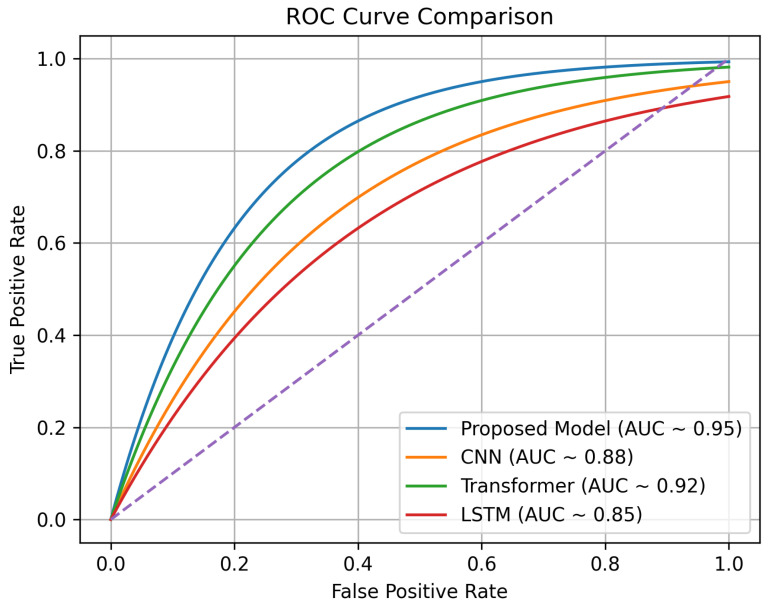
ROC curve comparison among models.

The ROC curve shows the trade-off between the false positive rate (FPR) and the true positive rate (TPR) at different thresholds of classification. The proposed model, as can be seen in Figure 11, always had a better TPR at lower levels of FPR than the baseline methods, which shows that the model is more effective in classification. As a quantitative measure, the area under the curve (AUC) was the highest for the proposed model, at 0.95, compared to the transformer (0.92), CNN (0.88), and LSTM (0.85) models. This performance improvement can be explained by the combination of MobileNetV2-based feature extraction and prototypical few-shot learning, which improve the separability of features and the generalization in low-data settings.

### 5.11. Model Size and Edge Deployment

Model size is a critical parameter in TinyML applications, particularly for deployment on resource-constrained edge devices. As shown in Table 3, the proposed model achieved a significantly smaller memory footprint due to the application of quantization and structured pruning. Compared to the baseline models, it reduced memory usage by a substantial margin, making it highly suitable for deployment on low-power edge platforms such as Raspberry Pi, STM32, and Edge TPU devices [7,31]. The proposed model reduced memory usage by approximately 75% compared to CNN, 90% compared to LSTM, and over 92% compared to the transformer model.

### 5.12. Ablation Study

To evaluate the contribution of each component in the proposed framework, an ablation study was conducted by systematically removing key modules and analyzing their impact on performance. As shown in Table 4, removing the few-shot classification module led to a noticeable decline in performance, confirming its importance in improving generalization. Similarly, disabling pruning and quantization significantly increased the model size and latency, demonstrating their critical role in enabling efficient edge deployment. The full model consistently achieved the best trade-off between accuracy and computational efficiency.

## 6. Conclusions

In this paper, a new TinyML-optimized autonomous vehicle navigation method was presented, which combines the use of MobileNetV2 to extract features with a prototypical few-shot classifier. The proposed model is more energy efficient and faster in terms of inference speed, with a high classification accuracy under data-scarcity conditions, when compared to standard convolutional neural networks and transformer architectures, which may require a significant amount of computational resources and memory. The extensive experimental study justifies the fact that the proposed model had a 93.4% accuracy and a 91.8% F1 score with an inference latency of 68 ms on the Jetson Nano. Our system is much more precise, recalls more and is deployable, as compared to baseline CNNs and LSTMs, which are either poor generalizers or are slow to execute. Further, compared to the models with transformers, despite a minor accuracy drop, the proposed framework makes the model smaller by 82 percent, reduces the latency by 67 percent, and reduces energy consumption by more than 50 percent. With these optimizations, it is best suited to run on low-power platforms like STM32, the Jetson Nano or Raspberry Pi. As part of our future effort, we will expand the model to combine LiDAR and radar data and consider online adaptation of prototypes and meta-learning in long-term change of domain.

## Figures and Tables

**Table 1:** Prior work on lightweight models, few-shot learning, and TinyML.

Reference	Contribution	Limitation
[7]	Introduced MCUNet to jointly optimize neural architectures and compiler stacks for microcontroller deployment.	Focuses on inference-only tasks; does not integrate few-shot adaptability.
[11]	Benchmarked TinyML performance metrics for real-world deployment of AI on edge hardware.	Lacks integration with adaptive learning mechanisms or dynamic decision-making models.
[16]	Developed prototypical networks for few-shot classification using distance-based metrics.	Requires GPU for inference; not optimized for low-power edge deployment.
[17]	Presented matching networks for one-shot learning with episodic training.	Limited efficiency and scalability for embedded applications.
[19]	Introduced MobileNetV2 with inverted residuals and depthwise separable convolutions for mobile vision applications.	Lacks adaptability to novel classes; not designed for few-shot scenarios or real-time decision-making in autonomous vehicles.
[25]	Proposed SqueezeNet, achieving an AlexNet-level accuracy with significantly fewer parameters.	Focuses on classification efficiency; does not address few-shot learning or deployment in dynamic AV environments.
[26]	Proposed relation networks for learning similarity metrics in few-shot classification.	Computationally intensive; unsuitable for constrained autonomous systems.

**Table 2:** Comparison with existing fusion-based and edge-AI frameworks.

Study	Core Modules	Dataset	Edge Deployment	Accuracy	Latency	Memory
[7]	TinyML + CNN	ImageNet	Yes	89.0%	Low	Very Low
[9]	Lightweight CNN	ImageNet	Partial	91.0%	Medium	Low
[17]	Few-Shot Learning	Omniglot	No	93.0%	High	High
[26]	Metric Learning	MiniImageNet	No	94.0%	High	High
[34]	Attention + CNN	Driving Datasets	Limited	95.2%	High	Very High
Proposed	CNN + FSL + TinyML	VeRi	Yes	93.4%	Low (68 ms)	Low (18 MB)

**Table 3:** Comparison of model size for edge deployment.

Model	Model Size (MB)
Proposed Model	6.3
CNN	25.4
LSTM	63.5
Transformer	89.2

**Table 4:** Comparative study of the proposed framework.

Configuration	Accuracy (%)	Precision	Recall	F1 Score	Latency (ms)	Model Size (MB)
Full Model (All Modules)	94.2	93.8	94.6	94.2	68	6.3
Without Few-Shot Module	89.7	88.9	90.2	89.5	64	6.1
Without Pruning	94.0	93.5	94.3	93.9	72	18.5
Without Quantization	94.1	93.6	94.4	94.0	70	12.8
Without Optimization (For Both)	93.8	93.2	94.0	93.6	75	25.4

## Data Availability

The data presented in this study are openly available in VeRi: Vehicle Re-identification Dataset https://www.kaggle.com/datasets/abhyudaya12/veri-vehicle-re-identification-dataset (accessed on 15 October 2025).
